# Psychotherapy for suicidal patients with borderline personality disorder: an expert consensus review of common factors across five therapies

**DOI:** 10.1186/2051-6673-1-16

**Published:** 2014-11-11

**Authors:** William Sledge, Eric M Plakun, Stephen Bauer, Beth Brodsky, Eve Caligor, Norman A Clemens, Serina Deen, Jerald Kay, Susan Lazar, Lisa A Mellman, Michael Myers, John Oldham, Frank Yeomans

**Affiliations:** Yale New Haven Psychiatric Hospital, 184 Liberty Street, New Haven, CT 06519 USA; Austen Riggs Center, Stockbridge, Massachusetts USA; Cornell University College of Medicine, New York, NY USA; Columbia University Medical Center, New York, NY USA; NYU School of Medicine, New York, NY USA; Case Western Reserve University, Cleveland, OH USA; University of California, San Francisco, CA USA; Boonshoft School of Medicine, Wright University, Dayton, OH USA; Georgetown University School of Medicine, Washington, DC USA; State University of New York, Brooklyn, NY USA; Baylor College of Medicine, The Menninger Clinic, Houston, TX USA; Weill Medical College of Cornell University, New York, NY USA

**Keywords:** Borderline Personality Disorder, Behavior therapy, Psychodynamic therapies, Common factors in psychotherapy

## Abstract

The objective was to review established literature on approaches to the psychotherapy of borderline personality disorder with specfic reference to suicide in order to determine if there were common factors across these efforts that would guide future teaching, practice and research.

The publications from the proponents of five therapies for the treatment of suicidal behavior in individuals with borderline personality disorder (BPD), were reviewed and discussed by the members of the Group for the Advanced of Psychiatry, Psychotherapy Committee (GAPPC). Twenty nine published research and summary reports were reviewed of the specific treatments noted above along with two other reviews of common factors for this group of treatments. We used expert consensus as to the salient articles for review and the appropriate level of abstraction for the common factor definition. We formulated a definition of effectiveness and identified six common factors: 1) negotiation of a specific frame for treatment, 2) recognition and insistence on the patient’s responsibilities within the therapy, 3) provision to the therapist of a conceptual framework for understanding and intervening, 4) use of the therapeutic relationship to engage and address suicide, 5) prioritization of suicide as a topic to be actively addressed whenever it emerges, and 6) provision of support for the therapist in the form of supervision, consultation or peer support. We discuss common factors, their formulation, and implications for development and teaching of psychotherapeutic approaches specific to suicide in patients with borderline personality disorder and note that there should be greater attention in practice and education to these issues.

## Introduction

Treating suicidal patients with borderline personality disorder (BPD) is a significant clinical challenge for mental health clinicians. Many clinicians have faced something like the situation described in the following vignette:

*A 23-year-old woman with BPD, in psychotherapy for the past 6 months with symptoms that include depression, cutting and suicidal ideation, is prescribed an antidepressant that has diminished her depression. She hints at the beginning of a session that after the session she intends to jump to her death.*

Clinicians react to such situations with anxiety, concern, anger, guilt, and worry about their patient as well their own legal liability, and often question whether they want to work with such risky and challenging patients. Moreover, the therapist’s strong reactions to the patient can lead to inaccurate estimations of risk and inappropriate treatment decisions.

## Review

Five explicitly articulated psychotherapies for suicidal BPD patients have significantly clear evidence of effective outcomes: Dialectical Behavior Therapy (DBT) [1], Schema Therapy (ST) [2], Transference Focused Psychotherapy (TFP) [3], Mentalization Based Therapy (MBT) [4], and Good Psychiatric Management (GPM) [5–8]. All are manualized, with published evidence of efficacy [1]. Two additional treatments reviewed by the committee used with Borderline Personality Disorder are included in an Appendix. They are Cognitive and Behavioral Therapy (CBT) [9] and Alliance Based Intervention for Suicide (ABIS) [10]. While the evidence is in conflict about effectiveness for suicide in one (CBT) [11] and not thoroughly studied in the other (ABIS), both are used robustly in a variety of settings and demonstrate promise.

Since relatively few clinicians are trained in even one of these specialized psychotherapies, we believe identification of common factors may prove useful to clinicians working with suicidal BPD patients and that awareness of common factors can also improve research, the development of new psychotherapies, and education.

### Indicators of effectiveness

We convened an expert panel of 13 clinicians and researchers composed of 12 psychiatrists and one clinical psychologist with experience teaching, conducting and investigating one or more of these six psychotherapies. Our expert panel, also the Psychotherapy Committee of the Group for the Group for the Advancement of Psychiatry (GAPPC), conceptualized three indicators of effectiveness for psychotherapeutic approaches to suicidality: 1) survival of the patient through control of suicidal behaviors; 2) keeping both therapist and patient engaged and committed to the psychotherapy; and 3) fostering the patient’s view of having a life worth living, an essential perspective of effective psychotherapy [12].

### Five therapies

Psychotherapies in general, particularly for BPD with suicidality, are mostly either behavioral or psychoanalytic in their theoretical ideology. Each takes different views of treatment based on fundamentally different theories about mental disorders and human nature. GPM integrates features from both of these perspectives [5–8].

### Behavior therapies

Behavioral therapies like DBT, and ST conceptualize therapy for suicidal behavior in patients with BPD as rectifying deficits in skills or capacities needed to tolerate intense affects and distortions in thinking.

#### Dialectical Behavior Therapy (DBT)

The DBT perspective [1, 8, 13–17] emphasizes the role of emotional dysregulation and impulsivity in suicide. DBT therapists offer skills training in 4 areas: distress tolerance, emotional regulation, interpersonal effectiveness, and mindfulness. DBT begins with a pre-treatment phase to forge a mutual commitment to eliminate suicidal behavior and entails an intensive approach with weekly individual therapy and skills training groups. Therapists meet weekly in consultative groups to help one another maintain a validating and “dialectic” stance toward their patients. The dialectic stance involves balancing validation strategies with change interventions.

DBT therapists would, as is true for most therapists practicing approaches addressed here, respond to the patient in the vignette by first assessing the patient’s suicide intent. As in other therapies, if intent to die were high, the therapist would engage the patient in revisiting her commitment to refrain from life-threatening behaviors and review plans for safety. If the patient is safe to proceed with therapy, the DBT therapist might explore whether and how her suicidal ideation is an expression of her distress. The DBT therapist might use an intervention called “extending” by taking the patient extremely seriously, possibly more seriously than the patient, and would wonder aloud about hospitalization with the expectation that the patient would back away from expressing suicidality in order to get a more desirable response, such as the therapist understanding her distress. This would allow the therapist to take the dialectic stance of validating the patient’s experience while engaging her in problem solving and developing a plan for using previously taught skills.

#### Schema Therapy (ST)

The clinical focus of ST is decreasing suicide risk by challenging negative thoughts and beliefs about oneself through cognitive techniques and behavioral experiments, while using the therapy relationship to improve the capacity to attach with others. ST employs “schema work,” helping the patient learn to identify 16 schemas -- beliefs about self in relation to others -- while encouraging positive schemas and discouraging negative ones. The therapist works for an alliance assuming the role of a novel healthy parent, with subsequent internalization of the therapist as healthy parent through “re-parenting” experiences, emotion focused work, cognitive restructuring and breaking behavioral patterns. ST sessions are twice weekly [18].

In the vignette the ST therapist would consider that the patient might be feeling like an abused, abandoned child (one of the schema modes), acting from the angry/impulsive child mode. The therapist might use experiential techniques such as imagery, role-playing, and letter writing to address suicidal thoughts and encourage skillful emotional expression. Behavioral techniques, such as relaxation, assertiveness training, anger management and gradual exposure to anxiety provoking situations, are used to help the patient gain control over emotions and behaviors toward the integration of these child-like schema modes into an effective adult mode.

### Psychodynamic therapies

Psychodynamic therapies approach suicide as substantially related to unconscious mental processes driving intense and unbearable affects. The task of psychodynamic therapies is to enable the patient to consciously grasp and gain mastery over these affects and processes, to use language and conscious thought - as opposed to action - to acknowledge, bear and integrate what was previously unconscious.

### Mentalization Based Therapy (MBT)

With an attachment perspective, MBT works toward improving the patient’s capacity to “mentalize,” that is, to keep in mind the patient’s own mind and the mind of the other [19–22]. Although a dynamic therapy, MBT avoids focusing on transference, free association, and fantasy, which are assumed to move the patient away from the capacity to mentalize, and does not pursue insight *per se*. MBT therapists view the patient as operating in a “psychic equivalence mode” based on rigid convictions, and use tactful self-disclosure to provide the patient a new perspective. MBT therapists focus on what the patient believes about a relationship rather than on the patterns behind it, and provides the patient with a written formulation of mentalization deficits to refer to throughout treatment and revise as necessary. MBT, as with most interventions, is intensive and usually carried out as once weekly individual sessions combined with group therapy.

In the vignette the MBT therapist would explore what might have occurred that led to the impulse to commit suicide and how that related to distress intolerance and the emergence of impulsive behavior instead of a fuller capacity to mentalize and bear the feelings without action. The therapist might refer to the written formulation to show the patient how the emergence of suicide in this moment represents a familiar vulnerability.

### Transference Focused Psychotherapy (TFP)

TFP employs a psychodynamic object relations model that includes structure, a clear frame, and limit setting [23–25]. TFP focuses on internal representations of self and other, and the affects that link them, as a way to understand the patient’s subjective and interpersonal experience. Suicide in BPD is understood as related to distorted images of self and others. TFP explores these internal representations and affect states as they relate to suicide with a focus on gaining awareness of the experience of self in relation to other that could motivate suicidal urges, ultimately creating the capacity for a stable, realistic and integrated experience of self and other in place of partial and extreme experiences of self and others. TFP sessions are twice weekly.

In the vignette a TFP therapist would review the initial agreement about the management of emergencies and explore with the patient the different perceptions of self and others that relate to the emergence of suicide, with their associated negative affects. The articulation and reflection on the patient’s extreme negative representations of self and other, and resolving the obstacles to linking these with positive representations experienced at other moments, would help the patient develop realistic, nuanced, and flexible views associated with moderate affects.

### Integrated psychotherapy

#### Good Psychiatric Management (GPM)

GPM is a model developed by Gunderson and Links [5–8] whose goal is to provide to all mental health professionals who treat BPD patients by psychotherapy basic knowledge necessary to treat this condition. It is usually a weekly individual therapy utilizing both psychodynamic and behavioral concepts. The treatment is described in terms of its distinctive characteristics (heavy reliance on case management, psychoeducation, goals, multimodal processes, duration, intensity). The “theory” that BPD is based on is interpersonal hypersensitivity.

The first basic principle of GPM is psychoeducation. A second principle is the persistent focus on the patient’s life outside therapy, linking the achievement of long-term life goals to the need to learn to control emotions or suicidality. A third principle is the therapist’s acknowledging and using his dual role as both a professional and a person. In the professional role, the therapist shares his/her knowledge, provides concerned but unemotional responses to a patient’s bursts of emotion and works to understand the patient’s recurring concerns about the therapist’s motives, feelings, and trustworthiness. The personal role (also known as the “real relationship in psychodynamic theory) is mobilized when the therapist explains what he/she meant when feeling misuderstood, discloses feelings, such as confusion or apprehension, and clearly states his/her wish to help. The fourth principle is the high level of the therapist’s responsiveness and activity compared with a traditional therapeutic approach. There is also a section on dealing with not liking the patient, which goes beyond the traditional idea of countertransference.

In this approach the therapist is relentlessly “pragmatic,” perhaps concrete and specific, and in addition to using psychoeducation, is active but thoughtful, real as a person and not distant as a professional, overtly expects change, is accountable and holds the patient accountable for all actions, focuses on the patient’s life outside therapy and is flexible. There is a clear agnostic sense that theory is relatively unimportant compared to pragmatic engagement of the issues, and that perspective, in a sense, becomes an over arching theory.

## Methodology

To determine whether these five psychotherapies met our effectiveness criteria and then to derive their common factors, we reviewed and compared the five approaches and considered the literature addressing their outcomes as well as their critical features. We examined the therapies through reading, review and discussion, and developed a consensus about common factors. We also discovered published citations [10, 26, 27] and unpublished investigations of common factors across evidence-based therapies for suicidal patients with BPD.

## Results

The committee reached consensus on six common factors that supported our definition of effectiveness:

### 1. Negotiation of a frame for treatment

All five approaches use a pre-treatment phase to clarify the role and responsibility of each participant before beginning treatment. This engages the patient as a collaborator with obligations and responsibilities that must be acknowledged for therapy to begin and continue. In the treatment of patients struggling with suicide, the pretreatment negotiation also includes identifying an explicit crisis plan in case the patient becomes unable to keep safe.

### 2. Recognition and insistence on the patient’s responsibilities within the therapy

All approaches agree on the requirement for explicitly shared recognition of the patient’s responsibilities within the treatment, along with the “real relationship,” the rational, reality based, socially sanctioned role and relationship between patient and therapist. Many patients respond to the recognition of their responsibility with appreciation of this affirmation of their competence as partners in the treatment endeavor. In instances where patients are not viewed as responsible for their safety, treatment approaches other than psychotherapy may be appropriate.

### 3. Provision to the therapist of a conceptual framework for understanding and intervening

The five approaches present a rational, internally consistent and cohesive model for clinician understanding of suicidal ideation and behavior, with a range of interventions that are intended to serve as mechanisms of change in suicidal patients. The concept of disorder, treatment philosophy and treatment process are aligned in a coherent, theoretical vision that can be shared and used in a specific manner to engage the suicidal patient and help the therapist maintain calm optimism in the face of stressful material.

### 4. Use of the therapeutic relationship to engage and address suicide actively and explicitly

Each approach recognizes the benefit of using the attachment with the therapist to engage and actively intervene with the symptom of suicide. The psychodynamic therapies focus on the transference meaning of suicide, that is, on feelings that can emerge in the patient during treatment that the therapist is indifferent, neglectful, or harmful to the patient. In behavioral treatments in which transference and the relationship itself are not emphasized, therapists use the relationship to provide support and to manage the contingencies of suicidal communications and behaviors. GPM repeatedly emphasizes the “realness” of the relationship (5).

### 5. Prioritization of suicide as a topic to be addressed whenever it emerges

All the approaches emphasize actively addressing suicidal thoughts and behaviors at the moment of their expression as a top priority. Nothing else can be more important in therapy than the threat to the life of the patient. While all approaches focus on suicidal thoughts and impulses, each conceptualizes the meaning of these differently.

### 6. Provision of support for the therapist

Explicit support to therapists working with suicidal BPD patients in the form of supervision, consultation, and/or peer support is also provided in all the approaches. While all psychotherapies support the value of ongoing consultation, peer review, and education, explicit support for the therapist is a particularly integral and essential component of approaches for working with suicidal patients.

## Discussion

When we focused on effectiveness goals we found six common factors among our group of six different effective psychotherapies. Others have pursued similar aims. The Boston Suicide Study Group (BSSG) [26] examined the manuals of five manualized therapies (four of those reported here). Their common “candidate interventions” across manuals included: 1) a clear treatment framework, 2) a defined strategy for managing suicide crises, 3) close attention to affect, 4) an active therapist style, and 5) the use of a theory of exploratory and change-oriented interventions in order to guide the treatment and ground it in an intellectual framework. Drawing from his research, Dr. Paul Links, in a discussion with Dr. William Sledge, defined “common principles” in evidence-based therapies for suicidal patients with BPD that included: 1) activity of the therapist to provide a stable framework, 2) therapist confidence based on a model of understanding, 3) empathic validation plus prioritization of addressing self destructive behaviors, 4) fostering “greater sense of self agency,” 5) connection between action and feelings, 6) differentiating lethal from non-lethal suicide intention, and 7) consultation and supervision for careful attention to countertransference [28]. Bateman also offered a set of “common characteristics,” including: 1) using a manual that supports the therapist and provides recommended interventions, 2) encouraging activity and self-agency for patients, 3) focusing on connections between action and feeling, 4) using psychoeduation to foster cognitive coherence in relation to subjective experience in the early phase of treatment, and 5) fostering an active stance by the therapist, including validation and empathy to generate a strong alliance [26].

Table 1 delineates areas of agreement between the GAPPC common factors, the BSSG candidate interventions (3 of the BSSG factors overlap with one or more of the GAPPC common factors), Links’ common principles (6 of his 7 principles overlap with one or more of the GAPPC common factors) and Bateman’s common characteristics (all of his 5 overlap with GAPPC common factors). Although there are differences in level of abstraction and emphasis (e.g., Links notes the importance of increased activity of the therapist to provide a stable framework, while GAPPC common factors emphasize the role of the pretreatment negotiation in providing a stable framework, and Bateman sees therapists as supported by the manual rather than by consultation meetings about manual adherence or by supervision or peer consultation), the degree of overlap in common factors from four independent sources suggests considerable agreement of their presence as common features of effective therapies for BPD patients struggling with suicide.

**Table 1:** **Comparison of treatments for borderline patients when suicide is the complaint**

Topic	GAPPC common factors	BSSG candidate interventions	Links common principles	Bateman common characteristics
Treatment structure	Negotiate a frame for treatment in pre-treatment phase to clarify responsibility and establish a crisis plan	Clear treatment framework.	Increased activity of the therapist to provide a stable framework	Active stance by the therapist to validate and demonstrate empathy, generate attachment and create alliance
		Defined strategy for managing suicide crises		
Patient role	Recognize and insist on patient’s responsibilities		Foster “greater sense of self agency”	Encourage increased activity, proactivity and self-agency for patient
Therapist behavior	Provide the therapist with coherent conceptualization of meaning of behavior and interventions	Use theory and associated interventions to guide and ground treatment	Promote confidence based on model of understanding.	Manual provides recommended interventions.
			Connection between action and feelings	Focus on connections between acts and feelings.
				Explain model of pathology to patient to promote cognitive coherence.
Therapeutic relationship	Use the therapeutic relationship and attachment to the therapist to engage suicide			Generate attachment, as above
Handling threat of suicide	Prioritize suicide as a topic to address in sessions		Empathic validation plus prioritization of self destructive behaviors	
Support for therapist	Provide support for the therapist		Consultation, supervision, attention to countertransference	Manual supports the therapist

There were areas of a lack of agreement. Bateman, Links and BSSG all make reference to increased activity of the therapist in such work. Although we did not emphasize this in our analysis, we believe this follows from implementation of the other common factors and we concur with the value. The BSSG candidate interventions and Bateman’s characteristics include reference to the importance of attending to the patient’s affect, while Links and Bateman both emphasize the connection between actions and feelings, points of view with which we agree. Links also notes a common principle of the importance of differentiating lethal from non-lethal suicide intention. This was not a common factor we agreed upon. It is not a view shared by DBT therapists, who view non-lethal, self-destructive and suicidal behavior on a continuum and thus to be engaged in the same way.

## Conclusions

Our study is a qualitative approach through expert consensus based on our experience and review of the literature. It is subject, therefore, to the caveats of qualitative research. Generalization from the few to the many is limited. A strength of our method is identification of hypotheses about practice. One hypothesis is that effective psychotherapy of suicidal patients, regardless of the underlying vision of the illness and its meaning or theory, contains factors that are common among them all and are necessary for effective treatment. We identified 5 effective, evidence based approaches that differed little in the content of six qualities of mid level of abstraction of the conduct of the therapy, but differed substantially from each other in theory and specific interactions. We believe that these core common factors are required for clinical effectiveness and that those seeking to develop, learn, teach or support psychotherapy for suicidality among patients with BPD, should ensure that these factors are present in their work.

## Appendix

This appendix includes treatments we reviewed that we believe have promise for effective treatments for BPD but either have conflicting evidence about effectiveness or have not been adequately investigated to make the claim outside the originating context. It is worth noting that these treatment approaches share the same common factors as the other five we studied and reported on above.

### Cognitive Behavioral Therapy (CBT)

The focus in CBT includes efforts to challenge negative and/or distorted automatic thoughts in order to decrease helplessness, while teaching and encouraging “behavioral activation interventions” to increase pleasure and mastery [29–31]. CBT has been modified to treat individuals with BPD so that the therapist attends to the emotions interfering with cognition through empathy, listening and the provision of support. In CBT dysfunctional beliefs of individuals with BPD include an impaired sense of their desirability because they see themselves as too needy, unlovable and/or deserving punishment, so they expect abandonment or loss of control and do not trust others. Sessions are generally weekly with homework.

In the vignette the CBT therapist would use empathic listening and expression of concern to calm the patient. Once the therapist determined that the patient was receptive, therapist and patient would collaborate to set a session agenda to identify the core dysfunctional beliefs or thoughts driving the patient’s current suicidal intent. The therapist might use thought records to restructure dysfunctional thinking, or behavioral and experiential techniques like role playing and experimenting with new behaviors.

### Alliance Based Intervention for Suicide (ABIS)

ABIS is an approach for establishing and maintaining a stable alliance in psychodynamic therapy with suicidal patients who are able to be committed to living [32]. Once suicide risk is reduced, the focus of therapy shifts to other problems in the patient’s life. Since a therapist practicing ABIS considers the murderousness of suicide to be part of the negative transference, s/he negotiates a therapeutic alliance that views suicide as representing destructive feelings toward the therapeutic work. When thoughts or actions related to suicide emerge, the ABIS therapist searches for their link to ruptures in the therapeutic relationship that may be explored and repaired. ABIS therapists use consultation to help manage intense countertransference related to work with suicidal patients. ABIS is part of psychodynamic therapy conducted once to four times weekly.

In the vignette the ABIS therapist would listen empathically to loss or distress that moved the patient toward suicide, and be curious about how this led the patient to consider ending the work to which they are both committed. Aware that suicide may be a response to emotional injury in the therapy, the therapist would explore whether the patient had recently perceived an injury, failure or rejection by the therapist, especially one that recreated in the transference relationship central past failures in relationships and search for how the emergence of suicide might represent a rupture between them that can be examined and repaired [6].

## Author’s information

William Sledge, MD is the Deputy Chair for Clinical Affairs and Program Development as well as Medical Director, of Yale New Haven Psychiatric Hospital. His clinical interests include psychotherapy; psychoanalytic psychotherapy and theory; hospital psychiatry; behavioral medicine; schizophrenia; and aviation psychiatry. His research interests include the topics of adaptation to chronic illness-physical and psychiatric, resilience and quality of life in special populations such as POWs and medically ill patients, quality improvement in medical care, and cost effectiveness in behavioral medicine programs. He is the George D and Esther S Gross Professor of Psychiatry at Yale School of Medicine.

Eric M. Plakun, MD is the Associate Medical Director and Director of Admissions of the Austen Riggs Center. He is a board certified psychiatrist, psychoanalyst, researcher and forensic psychiatrist. Dr. Plakun is the editor of New Perspectives on Narcissism (American Psychiatric Press, 1990) and Treatment Resistance and Patient Authority: The Austen Riggs Reader (Norton Professional Books, 2011), and author of more than thirty-five articles and book chapters on the diagnosis, treatment, longitudinal course and outcome of patients with borderline personality disorder and treatment resistant disorders.

(all else in alphabetical order by last name)

Stephen Bauer, MD is Associate Clinical Professor Emeritus, Weill Cornell Medical College and Senior Lecturer, Columbia University College of P&S, and has had a long standing interest in the diagnosis and treatment of severely ill psychiatric patients and was part of the group that developed Transference Focused Therapy for Borderline Conditions. He has published in these areas.

Beth Brodsky, PhD is Associate Clinical Professor of Medical Psychology in Psychiatry at Columbia University, and a research scientist in the Silvio O. Conte Center for the Neurobiology of Mental Disorders, at the New York State Psychiatric Institute, Department of Molecular Imaging and Neuropathology. Her areas of expertise include research and psychotherapeutic treatment of self-destructive behavior in borderline personality disorder (BPD). She has published numerous articles and chapters on the topics of suicidal risk and treatment of suicidal behavior in borderline personality disorder. She is trained in Dialectical Behavior Therapy and teaches DBT to psychiatry residents and psychology trainees.

Eve Caligor, MD is Clinical Professor and Associate Director of Residency Training for the Department of Psychiatry at New York University’s School of Medicine. Dr. Caligor's primary clinical and research interests are the evaluation and treatment of personality disorders. As an educator, Dr. Caligor is intrigued by the possibility of applying her clinical and research expertise to residency education and psychotherapy training.

Norman A. Clemens, MD, is Emeritus Clinical Professor of Psychiatry at Case Western Reserve University School of Medicine, a Training and Supervising Analyst at the Cleveland Psychoanalytic Center, and a member of the Board of Regents of the American College of Psychoanalysts.

Serina Deen, MD is Health Sciences Assistant Clinical Professor at the University of California, San Francisco. Her areas of interest include mindfulness, medical education, writing for the lay public, and psychotherapy.

Jerald Kay MD, is Professor and Chair of Psychiatry at the Wright University Boonshoft School of Medicine. His areas of interest include, the integration of psychotherapies, biologic dimensions of psychotherapy, and community organization of psychiatric services.

Susan Lazar, MD is a Clinical Professor of Psychiatry at Georgetown University School of Medicine, George Washington University School of Medicine, and the Uniformed Services University of the Health Sciences. She is a supervising and training analyst at the Washington Psychoanalytic Institute. She is the coauthor and editor of Psychotherapy Is Worth It: A Comprehensive Review of Its Cost-Effectiveness from American Psychiatric Publishing.

Lisa A. Mellman, M.D. is Clinical Professor of Psychiatry and Senior Associate Dean for Student Affairs at Columbia University College of Physicians & Surgeons. She is Past President of the American Association of Directors of Psychiatry Residency Training and chaired task forces and committees on psychiatric education and psychotherapy competency for the American Psychiatric Association and the American Association of Directors of Psychiatry Residency Training. She also chaired the Fellowship Program of the American Psychoanalytic Association.

Michael Myers, MD is Professor of Clinical Psychiatry and immediate past Vice-Chair of Education and Director of Training in the Department of Psychiatry & Behavioral Sciences at SUNY-Downstate Medical Center. Along with his continuing clinical research, teaching and outreach in the field of suicide, Dr Myers is the immediate past President of the New York City Chapter of the American Foundation for Suicide Prevention. He also serves on the Clinician Survivor Task Force of the American Association of Suicidology and is a frequent presenter at their annual meetings.

John Oldham, MD currently holds the positions of Senior Vice President and Chief of Staff at The Menninger Clinic, and Professor at the Menninger Department of Psychiatry & Behavioral Sciences, at Baylor College of Medicine. Dr. Oldham is senior editor of the Textbook of Personality Disorders and the Essentials of Personality Disorders. He was also a member of the Personality and Personality Disorders Workgroup, which made recommendations for changes to the fifth edition of the Diagnostic and Statistical Manual of Mental Disorders.

Frank Yeomans, MD is Clinical Associate Professor of Psychiatry at the Weill Medical College of Cornell University, Director of Training at the Personality Disorders Institute of Weill-Cornell, at the Columbia University College of Physicians and Surgeons Center for Psychoanalytic Training and Research, and Director of the Personality Studies Institute in Manhattan.

## Abbreviations

ABIS: Alliance Based Intervention for Suicide
BPD: Borderline Personality Disorder
BSSG: Boston Suicide Study Group
CBT: Cognitive Behavior Therapy
DBT: Dialectical Behavior Therapy
GAPPC: Group for the Advanced of Psychiatry, Psychotherapy Committee
GPM: Good Psychiatric Management
MBT: Mentalization Based Therapy
ST: Schema Therapy
TFP: Transference Focused Psychotherapy.
